# Risk Factors for Coronary Artery Lesionsin Kawasaki Disease Independent of Antibiotic Use in Chinese Children

**DOI:** 10.3389/fpubh.2022.817613

**Published:** 2022-05-05

**Authors:** Sixian Lao, Tao Zhou, Ho-Chang Kuo, Guoping Zhong, Weiwei Zeng

**Affiliations:** ^1^Institute of Clinical Pharmacology, School of Pharmaceutical Sciences, Sun Yat-sen University, Guangzhou, China; ^2^The Second People's Hospital of Longgang District, Shenzhen, China; ^3^Shenzhen Baoan Women's and Children's Hospital, Jinan University, Shenzhen, China; ^4^Guangdong Provincial Key Laboratory of New Drug Design and Evaluation, Guangzhou, China; ^5^Kawasaki Disease Center and Department of Pediatrics, Kaohsiung Chang Gung Memorial Hospital, Kaohsiung, Taiwan; ^6^College of Medicine, Chang Gung University, Taoyuan, Taiwan

**Keywords:** Kawasaki disease, coronary artery lesions, risk factors, antibiotics, children

## Abstract

**Objectives:**

To study the impact of antibiotics used in Kawasaki disease (KD) with coronary artery lesions (CAL) and identify independent risk factors.

**Methodology:**

This study reviewed the records of 287 KD patients between the years 2016 and 2020. Patients were grouped by their outcome, the CAL group, and a no-coronary artery lesions (NCAL) group, and stratified by the use of antibiotics. We collected clinical and laboratory data before the intravenous immunoglobulin (IVIG) treatment.

**Results:**

The two groups of KD patients with and without CAL were compared. The results showed that there are significant differences between groups which were erythrocyte count (p = 0.045) and hemoglobin (p = 0.005), red blood cell-specific volume (p = 0.001), immature granular cells percentage (p = 0.006), total protein (p = 0.045), albumin (p = 0.041), alkaline phosphatase (p = 0.023), and chlorine (p = 0.006). After multivariate logistic regression, neutrophil granulocyte percentage (odds ratio [OR] = 1.200, 95% confidence interval [CI]: 1.008-1.428, p = 0.040), lymphocyte percentage (p = 0.028, OR = 1.243, 95% CI: 1.024-1.508, p = 0.028) and total protein (OR = 4.414, 95% CI: 1.092-17.846, p = 0.037) were found to be independent risk factors for CAL. After analyzing the cases with a history of antibiotic use, multivariate analysis showed no indicators were considered independent risk factors for CAL.

**Conclusion:**

Neutrophil granulocyte percentage, Lymphocyte percentage and total protein were independent risks for CAL in KD without antibiotics use history. The use of antibiotics affected physiological indicators of KD patients.

## Introduction

Kawasaki disease (KD) is an afebrile systemic vasculitis disease, which was first termed mucocutaneous lymph node syndrome (MCLS) after it was first described in Japan in 1967 (1). It is a form of severe vasculitis of the arteries that leads to an increased risk of atherosclerosis and ischemic heart disease (2), especially inducing coronary artery lesions (CAL). In infants and young children, the data published by the Japanese KD National Survey reported that over time, the rate has risen from 218.6 per 100,000 in 2008 to 330.2/10 million in 2015. Approximately 4,000 to 5,500 new cases are added in the United States each year (3), making it the leading cause of acquired heart disease in children (4–6). Multiple studies have shown that CAL, or aneurysm formation, is the most common and most important clinical complication of KD. The 23rd National Survey of Japan in 2015–2016 reported that the cardiac lesion rate of KD was 11.9%, with most of them having acoronary complications (7). Between 2006 and 2012, the incidence of CAL in KD patients in Shanghai was 15.9% (8), and 9.1% between 2013 and 2017 (9). From 2011 to 2016, CAL was the most common finding (23%) in Spain (10). Coronary dilatation occurred in 10.8% of the patients in South Korea in 2012–2014 (11). Among Australian KD patients, 16.7% of them had coronary artery ectasia or dilation from 1979 to 2009 (12). Finally, 12.9% of the patients had CAL in the USA from 1994–2003 (13).

The standard treatment for KD is a combination of intravenous immunoglobulin (IVIG) and acetylsalicylic acid (ASA) (5, 14, 15). Early and timely administration of this regimen will help improve the treatment response and lower the risk of CAL (6, 16).

KD must be identified between 4 and 9 days after presenting with acute fever. But the early clinical symptoms of KD are not obvious, and it is often misdiagnosed as a simple fever disease in the clinic. A delayed diagnosis of KD will increase the cost of treatment, increase the risk of CAL, and result in inpatients receiving ineffective drugs such as antibiotics. Antibiotics are particularly ineffective for KD patients (5) but limited by the difficulty of clinical diagnosis.

In recent years, methods to identify patients who are more likely to have CAL have attracted significant research. Clarification of the risk factors of KD with CAL from biochemical indicators and establishing a scoring system is essential (1) and will allow active intervention and the development of follow-up strategies. Several scoring models for predicting CAL development have been proposed previously (1, 4, 6, 17–19), but the models differ by location and ethnicity, leading to different scoring systems.

None of the previous studies took into account the impact of antibiotics on the patient's biological indicators.However, in children, non-essential antibiotics may cause adverse effects such as rash, eosinophilia, fever, and pancytopenia (20), but also alter the gut microbial composition, affecting the patient's immunity, metabolism, and endocrine system (21). While the use of antibiotics can lead to health complications in KD patients, it can also change the patient's biochemical indicators.

This study compared the groups with or without antibiotic treatment for KD to explore the impact of antibiotics on the diagnosis of CAL in KD.

## Methods

We reviewed retrospective data of KD patients at the Baoan Women's and Children's Hospital in Shenzhen, China, between 2016 and 2020. All patients were younger than 12 years old when they were diagnosed with KD in the analyzed data. The studies have been approved by the Institutional Review Board of Baoan Women's and Children's Hospital, Shenzhen, China with IRB No. LLSCHY2021-1-07. This study was performed under the Declaration of Helsinki.

### Definitions of Kawasaki Disease and Coronary Artery Lesion

The diagnosis of KD was made according to the American Heart Association criteria (5). To make a KD diagnosis, the patient must have a fever lasting more than 5 days and have at least four of the following five symptoms: bulbous conjunctival congestion, lips or oral cavity changes, hand and foot symptoms (peripheral edema, peripheral erythema, periungual desquamation), skin manifestations, and cervical lymph node enlargement (22).

The arteries were assessed with an echocardiogram. CAL or aneurysm formation was defined by age-specific internal lumen diameter: ≥2.5 mm in patients younger than 3 years, >3 mm in patients aged 3–9 years, and >3.5 mm in patients aged 9–14 years. Additionally, the internal diameter of a segment was measured ≥1.5 times that of an adjacent segment, or the lumen was clearly irregular (23).

### Statistical Analysis

All analyses were performed using SPSS version 14.0 (IBM, Armonk, NY). After testing the normality of continuous variables or the number of cases and percentage for categorical variables, data were analyzed using the median (Q1–Q3). We used the Mann-Whitney *U*-test, or Fisher's exact test, to compare the laboratory test results between those with and without CAL. Univariate logistic regression was used to analyze the influence of variables on CAL. By multivariate logistic regression analysis, variables were used to determine the independent risk factors for CAL. The odds ratio (OR) and 95% confidence interval (CI) were calculated in the logistic regression analysis. The *p*-value < 0.05 was considered statistically significant.

## Results

### Patients

We analyzed the data of a total of 287 KD patients, including 191 males and 96 females. Overall, there were 38 patients with CAL formation (13.2%). Compared to females (8.3%), a higher percentage of males (15.7%) had a CAL, but this finding was not significantly different (*p* = 0.097).

### Risk Factors for CAL in All Patients

Univariate analysis showed that C reactive protein (CRP) (CAL: 20.60 vs. NCAL: 58.40 mg/L *p* = 0.005), duration of fever, (CAL: 4.00 vs. NCAL: 5.00 day *p* = 0.017), hemoglobin (CAL: 111.00 vs. NCAL: 108.00 × 10^9^/*L* *p* = 0.043), erythrocyte count (CAL: 34.50 vs. NCAL: 32.45 × 10^9^/*L*, *p* = 0.007), albumin (CAL: 38.40 g/L vs. NCAL:37.00 g/L, *p* = 0.040), alkaline phosphatase (CAL:190.00 U/L vs. NCAL: 220.00 U/L, *p* = 0.013), and chlorine (CAL:103.80 mmol/L vs. NCAL: 101.55 mmol/L, *p* = 0.002) showed significant differences between the two groups (Table 1).

**Table 1:** Comparison of groups with and without coronary artery lesions.

**Variables**	**NCAL**		**CAL**		**Univariate**	**Multivariable(all)**			
	***n***	**Median (Q1–Q3)**	***n***	**Median (Q1–Q3)**	***P_**1**_***	***P_**2**_***	**OR**	**95%CI**	
								**Lower**	**Upper**
Male gender (%)	161	64.7%	30	78.9%	0.097	0.087	2.050	0.901	4.663
Age, year	249	2.25(1.33–3.92)	38	2.13(1.06–3.67)	0.498	0.565	1.014	0.968	1.062
Bodyweight, kg	248	12.00(9.53–15.00)	38	12.60(10.00–15.33)	0.314	0.849	1.017	0.852	1.216
CRP, mg/L	217	58.40(31.51–101.46)	31	20.60(0.80–85.01)	**0.005**	0.421	0.997	0.989	1.005
Duration of fever, day	248	5.00(3.00–6.00)	38	4.00(0.00–5.00)	**0.017**	**0.004**	0.766	0.639	0.919
White blood cell count, × 10^9^/L	242	11.69(8.50–16.34)	37	12.50(9.20–14.47)	0.589	0.650	0.967	0.839	1.116
Red blood cell specific volume, %	237	4.14(3.85–4.48)	37	4.33(3.89–4.80)	0.102	0.638	1.289	0.448	3.705
Hemoglobin, g/L	241	108.00(99.50–115.00)	37	111.00(103.00–121.50)	**0.043**	0.681	0.991	0.950	1.034
Platelet count, × 10^9^/L	242	419.00(320.50–528.00)	37	426.00(376.00–544.50)	0.223	0.604	0.999	0.995	1.003
Erythrocyte count, × 10^9^/L	244	32.45(30.10–35.00)	37	34.50(31.50–37.10)	**0.007**	0.486	0.958	0.850	1.080
Neutrophil granulocyte percentage, %	243	51.40(37.3–66.30)	37	53.40(34.50–62.00)	0.873	0.487	1.183	0.736	1.903
Lymphocyte percentage, %	242	34.30(22.58–49.48)	37	35.70(27.95–51.35)	0.358	0.455	1.197	0.747	1.918
Monocyte percentage, %	241	6.50(4.90–8.90)	37	7.00(4.60–10.05)	0.644	0.617	1.123	0.713	1.771
Eosinophilic granulocyte percentage, %	241	2.10(0.50–4.90)	37	1.90(0.40–4.35)	0.680	0.636	1.128	0.685	1.855
Basophile percentage, %	241	0.30(0.20–0.50)	37	0.30(0.20–0.50)	0.486	0.832	1.377	0.072	26.224
Immature granular cells percentage, %	241	0.50(0.30–1.40)	34	0.45(0.20–2.25)	0.899	0.018*	1.310	1.048	1.638
Total protein, g/L	211	66.00(62.00–70.00)	30	67.20(63.83–70.25)	0.261	0.016*	2.573	1.191	5.561
Albumin, g/L	211	37.00(34.00–39.90)	30	38.40(36.00–42.25)	**0.040**	**0.028**	0.419	0.193	0.911
Globulin, g/L	211	28.50(25.30–32.00)	30	27.50(24.30–30.85)	0.226	**0.022**	0.408	0.190	0.877
Total bilirubin, μmol/L	210	6.60(4.80–9.30)	30	5.20(3.75–8.60)	0.100	0.388	0.961	0.877	1.052
Alanine aminotransferase, U/L	210	19.75(12.00–46.43)	30	17.00(13–35.73)	0.761	0.710	1.003	0.988	1.018
Aspartate transaminase, U/L	211	33.00(27.00–46.00)	30	37.00(31.50–50.75)	0.067	0.633	0.995	0.973	1.017
Alkaline phosphatase, U/L	210	190.00(152.00–235.25)	30	220.00(179.75–296.00)	**0.013**	0.680	1.001	0.998	1.003
Creatinine, μmol/L	200	25.00(21.00–30.00)	29	24.00(22.50–31.00)	0.795	0.645	0.982	0.907	1.063
Urea, mmol/L	198	3.00(2.48–3.70)	29	3.40(2.60–4.10)	0.132	0.674	0.989	0.941	1.040
Uric acid, μmol/L	199	220.00(179.00–280.00)	29	242.00(215.00–298.00)	0.151	0.492	1.002	0.996	1.009
Potassium, mmol/L	212	4.31(4.01–4.77)	31	4.40(3.92–4.85)	0.905	0.926	1.054	0.350	3.174
Sodium, mmol/L	212	136.00(134.00–138.00)	31	137.00(135.00–138.00)	0.114	0.653	0.938	0.708	1.242
Chlorine, mmol/L	212	101.55(99.23–104.00)	31	103.80(101.00–105.00)	**0.002**	0.024*	1.325	1.038	1.692
Calcium, mmol/L	212	2.30(2.21–2.41)	31	2.37(2.27–2.46)	0.094	0.667	0.423	0.008	21.316
Magnesium, mmol/L	212	0.89(0.84–0.95)	31	0.89(0.85–0.95)	0.933	0.915	1.468	0.001	1688.702

NCAL, non-coronary artery lesions; CAL, coronary artery lesions; P_1_, P of univariate; P_2_, P of multivariable. *p < 0.05.

Multivariate logistic regression, immature granular cell percentage (*p* = 0.018, OR = 1.310, 95% CI: 1.048–1.638), total protein (*p* = 0.016, OR = 2.573, 95%CI: 1.191–5.561) and chlorine (*p* = 0.024, OR = 1.325, 95%CI: 1.038–1.629) were considered as the independent risk factors for CAL formation in KD patients without antibiotic usage (Table 1).

### Risk Factors for CAL in Patients Without Using Antibiotic

Two hundred and ten patients didn't receive antibiotic treatment before being diagnosed with KD, including 134 males (63.8%) and 76 females (36.2%). The CAL group contained 28 patients, and the non-CAL (NCAL) group consisted of 182 patients.Gender (*P* = 0.296), age (*P* = 0.688), and body weight (*P* = 0.103) were not significantly associated with CAL formation in this subgroup analysis.

Nine variables, including C reactive protein, the duration of fever, erythrocyte count, hemoglobin, red blood cell-specific volume, immature granular cell percentage, total protein, albumin, alkaline phosphatase, and chlorine, showed significant differences between the two groups (as shown in Table 2). After multivariate logistic regression, immature granular cell percentage (*p* = 0.006, OR = 1.417, 95% CI: 1.103–1.819), and total protein (*p* = 0.022, OR = 3.193, 95% CI: 1.182–8.624) were considered as the independent risk factors for CAL formation in KD patients without antibiotic usage (Table 2).

**Table 2:** Comparison of groups with and without coronary artery lesions before the use of antibiotics.

**Variables**	**NCAL**		**CAL**		**Univariate**	**Multivariable (all)**			
	***n***	**Median (Q1-Q3)**	***n***	**Median (Q1-Q3)**	***P_**1**_***	***P_**2**_***	**OR**	**95%CI**	
								**Lower**	**Upper**
Age, year	182	2.08(1.25–3.60)	28	2.21(1.25–3.84)	0.688	0.572	1.013	0.968	1.061
CRP, mg/L	168	66.02(34.59–106.14)	24	6.50(0.60–79.51)	**0.003**	0.403	0.996	0.987	1.005
Duration of fever, day	182	5.00(3.00–6.00)	28	4.00(0.00–5.00)	**0.006**	**0.009**	0.756	0.612	0.934
Bodyweight, kg	182	11.85(9.30–15.00)	28	13.25(10.00–15.75)	0.103	0.632	1.050	0.860	1.283
White blood cell count, × 10^9^/L	175	12.13(8.90–17.00)	27	12.87(9.32–14.48)	0.539	0.718	0.972	0.835	1.132
Erythrocyte count, × 10^9^/L	170	4.15(3.87–4.47)	27	4.42(3.99–4.80)	**0.045**	0.380	1.688	0.525	5.430
Hemoglobin, g/L	174	108.00(99.00–115.00)	27	114.00(105.00–125.00)	**0.005**	1.000	1.000	0.950	1.052
Platelet count, × 10^9^/L	175	423.00(327.00–528.00)	27	423.00(389.00–538.00)	0.293	0.545	0.999	0.995	1.003
Red blood cell specific volume, %	177	32.25(30.00-32.60)	27	35.00(33.00–37.80)	**0.001**	0.339	0.939	0.824	1.069
Neutrophil granulocyte percentage, %	176	55.35(37.95–68.60)	27	53.40(32.20–61.70)	0.327	0.415	1.237	0.742	2.061
Lymphocyte percentage, %	175	31.50(21.10–48.50)	27	39.40(29.80–52.80)	0.090	0.356	1.269	0.765	2.106
Monocyte percentage, %	174	6.30(4.68–8.70)	27	7.00(5.30–10.10)	0.251	0.634	1.125	0.692	1.831
Eosinophilic granulocyte percentage, %	174	2.20(0.50–5.03)	27	2.00(0.80–4.10)	0.914	0.711	1.113	0.633	1.956
Basophile percentage, %	174	0.30(0.20–0.50)	27	0.40(0.20–0.50)	0.183	0.357	5.159	0.158	168.930
Immature granular cells percentage, %	175	0.50(0.30–1.00)	26	0.55(0.20–4.00)	0.397	0.006*	1.417	1.103	1.819
Total protein, g/L	169	65.00(62.00–69.05)	26	67.40(64.15–70.25)	**0.045**	0.022*	3.193	1.182	8.624
Albumin, g/L	169	37.70(34.00–39.95)	26	38.80(356.38–43.00)	**0.041**	**0.031**	0.335	0.124	0.905
Globulin, g/L	169	28.00(25.05–31.15)	26	28.40(24.85–30.85)	0.789	**0.031**	0.336	0.124	0.906
Total bilirubin, μmol/L	168	6.60(4.93–9.80)	26	5.20(3.75–10.45)	0.132	0.853	0.989	0.883	1.109
Alanine aminotransferase, U/L	168	19.90(12.00–48.15)	26	16.50(13.00–35.73)	0.695	0.927	0.999	0.980	1.019
Aspartate transaminase, U/L	169	33.00(26.50–46.50)	26	36.50(31.50–50.75)	0.095	0.547	0.992	0.966	1.019
Alkaline phosphatase, U/L	168	193.50(159.25–241.00)	26	222.50(186.00–296.50)	**0.023**	0.983	1.000	0.997	1.003
Creatinine, μmol/L	165	25.00(21.00–30.00)	26	24.00(21.75–30.25)	0.906	0.553	0.968	0.871	1.077
Urea, mmol/L	164	3.10(2.50–3.80)	26	3.30(2.45–4.23)	0.313	0.751	0.987	0.909	1.071
Uric acid, μmol/L	165	222.00(180.00–284.50)	26	240.50(201.75–280.00)	0.437	0.776	0.999	0.991	1.007
Potassium,mmol/L	169	4.30(4.03–4.72)	26	4.40(3.92–4.75)	0.975	0.579	1.399	0.427	4.577
Sodium,mmol/L	169	136.00(134.00–138.00)	26	–	0.152	0.515	0.902	0.662	1.230
Chlorine,mmol/L	169	101.80(99.10–104.00)	26	103.80(101.00–105.83)	**0.006**	0.077	1.279	0.974	1.679
Calcium,mmol/L	169	2.32(2.22–2.42)	26	2.37(2.30–2.47)	0.071	0.957	1.121	0.017	72.910
Magnesium,mmol/L	169	0.90(0.85–0.96)	26	0.90(0.86–0.94)	0.764	0.883	0.540	0.000	1988.484

NCAL, non-coronary artery lesions; CAL, coronary artery lesions; P_1_, P of univariate; P_2_, P of multivariable. *p < 0.05.

### Risk Factors for CAL in Patients With Antibiotic Use

Univariate analysis showed that there were no significant differences in the clinical indicators between these two groups. Variables with subjects *N* ≥ 10 were included for multivariate analysis and revealed that no independent indicator was considered for predicting CAL formation in KD patients (Table 3).

**Table 3:** Comparison of groups with coronary artery lesions and no-coronary artery lesions with using anibiotics.

**Variables**	**NCAL**		**CAL**		**Univariate**	**Multivariable (all)**			
	***n***	**Median (Q1-Q3)**	***n***	**Median (Q1-Q3)**	***P_**1**_***	***P_**2**_***	**OR**	**95%CI**	
								**Lower**	**Upper**
Age (year)	67	2.75(1.75–4.92)	10	1.05(0.76–3.60)	0.071	**0.033**	0.258	0.074	0.897
CRP, mg/L	49	42.56(23.00–76.85)	7	26.42(20.60–92.56)	0.785	0.978	1.000	0.985	1.014
Duration of fever, day	66	4.00(3.00–6.00)	10	5.00(2.00-7.00)	0.975	0.228	0.819	0.567	1.183
Bodyweight (kg)	67	12.50(10.00–17.00)	10	10.25(9.07–15.83)	0.503	0.101	1.392	0.937	2.068
White blood cell count, × 10^9^/L	67	10.30(7.00–14.50)	10	9,78(8,71–14.70)	0.994	0.096	0.809	0.631	1.038
Erythrocyte count, × 10^9^/L	67	4.03(3.72–4.48)	10	3.93(3.83–4.46)	0.862	0.633	1.610	0.228	11.365
Hemoglobin, g/L	67	105.00(100.00–114.00)	10	103.00(97.75–107.25)	0.539	0.942	1.003	0.920	1.093
Platelet count, × 10^9^/L	67	410.00(272.00–526.00)	10	465.00(300.25–722.75)	0.495	0.247	1.002	0.999	1.005
Red blood cell specific volume, %	67	32.30(30.80–34.50)	10	32.00(29.58–35.25)	0.814	0.499	1.081	0.862	1.356
Neutrophil granulocyte percentage, %	67	47.80(37.30–64.10)	10	46.35(33.05–55.18)	0.405	0.065	0.809	0.646	1.014
Lymphocyte percentage, %	67	37.20(24.00–51.60)	10	41.60(31.50–57.43)	0.336	0.098	0.820	0.649	1.037
Monocyte percentage, %	67	8.00(5.50–9.80)	10	6.30(5.58–8.43)	0.347	0.159	0.795	0.577	1.094
Eosinophilic granulocyte percentage, %	67	1.70(0.40–.40)	10	3.00(1.03–8.95)	0.268	0.833	1.010	0.923	1.104
Basophile percentage, %	67	0.30(0.20–0.40)	10	0.30(0.20–0.55)	0.450	0.653	2.550	0.043	151.029
Immature granular cells percentage, %	67	0.40(0.03–1.08)	10	0.85(30.38–5.21)	0.125				
Total protein	42	68.50(63.18–73.80)	4	63.30(53.15–70.98)	0.226				
Albumin	42	35.00(32.00–38.50)	4	33.60(27.20–41.05)	0.953				
Globulin	42	30.95(26.00–41.25)	4	22.75(21.00–41.15)	0.118				
Total bilirubin	42	6.65(4.58–8.83)	4	5.80(3.38–7.48)	0.520				
Alanine aminotransferase	42	19.00(12.93–45.25)	4	18.00(13.10–119.20)	0.861				
Aspartate transaminase	42	34.50(28.00–45.00)	4	42.00(30.50–71.50)	0.369				
Alkaline phosphatase	42	156.74(123.75–198.75)	4	179.50(135.25–265.00	0.559				
Potassium	34	4.32(3.99–4.93)	5	4.37(4.01–4.87)	0.893				
Sodium	43	136.00(133.00–137.19)	5	135.00(134.50–138.70)	0.564				
Chlorine	43	101.00(99.70–1-2.70)	5	103.80(101.40–104.00)	0.100				
Calcium	43	2.27(2.20–2.40)	5	2.22(2.11–2.45)	0.761				
Magnesium	43	0.88(0.78–0.92)	5	0.85(0.82–1.06)	0.457				

*NCAL, non coronary artery lesions; CAL, coronary artery lesions; P_1_, P value of univariate; P_2_, P value of multivariable*.

### Antibiotic Treatment Compared to No Antibiotic Treatment

A total of 77 patients received antibiotic treatment, while 210 KD patients received no antibiotics during the study period. There were no significant differences between the two groups in gender (*p* = 0.121) or age distribution (*p* = 0.102). There were significant differences in albumin (*p* = 0.004) and globulin (*p* = 0.005) between the two groups (Table 4).

**Table 4:** Comparison of groups with antibiotic and No-antibiotic.

	**Variables**	**Antibiotic (*****n*** **=** **210)**		**No-antibiotic (*****n*** **=** **77)**		***P***
		***n***	**Median (Q1–Q3)**	***n***	**Median (Q1–Q3)**	
All cases (*n* = 287)	Immature granular cells percentage, %	63	0.20(0.01–0.90)	186	0.05(0.01–0.50)	0.051
	Total protein, g/L	46	68.00(62.20–73.33)	195	65.60(62.10–69.60)	0.074
	Albumin, g/L	46	35.00(32.00–38.65)	195	37.80(34.20-40.00)	**0.004**
	Globulin, g/L	46	30.80(26.00–41.25)	195	28.00(25.00–31.00)	**0.005**
	Chlorine,mmol/L	48	101.13(100.0–103.00)	195	102.00(99.60–104.20)	0.434
Without CAL (*n* = 249)	Immature granular cells percentage, %	66	0.60(0.23–2.25)	175	0.50(0.30–1.00)	0.294
	Total protein, g/L	42	68.50(63.18–73.80)	169	65.00(62.00–69.05)	**0.019**
	Albumin, g/L	42	35.00(32.00–38.50)	169	37.70(24.00–39.95)	**0.015**
	Globulin, g/L	42	30.95(26.00–41.25)	169	28.00(25.05–31.15)	**0.001**
	Chlorine, mmol/L	43	101.0099(70–102.70)	169	101.80(99.10–104.00)	0.563

*CAL, coronary artery lesions*.

After excluding the cases with CAL, total protein (*p* = 0.019), albumin (*p* = 0.015), and globulin (*p* = 0.001) were significant differences between the two groups. Among them, the antibiotic group showed higher total protein (68.50 vs. 65.00 g/L) and higher globulin (30.95 vs. 28.00 g/L), but lower albumin (35.00 vs. 37.70 g/L) (Table 4).

## Discussion

In our study, 38 patients were diagnosed with CAL or aneurysm formation (13.2%). Compared to females (8.3%), a higher percentage of males (15.7%) had a CAL. The percentage of males and females with CAL didn't show significant differences (*p* = 0.097). The data included in this study shows that the incidence of CAL is slightly lower than other reports (24). This is consistent with the difference in the incidence of KD and CAL in different regions. The prevalence of CAL in males is higher than that in females, indicating that gender may be one of the factors leading to the occurrence of CAL. Being a boy was considered an independent risk factor for KD combined with CAL in related studies (1).

The major targeted tissue in KD-related vasculitis tends to be the coronary artery, and this has attracted extensive attention from pediatricians. Early identification of risk factors for an accurate prediction of CAL or aneurysm formation continues to be the focus of the current studies, however, the reported scoring system has the problem of low predictive power in different samples (24). Because the diagnostic criteria of KD include clinical features common to other childhood febrile diseases. KD is sometimes confused with bacterial infections, leading to KD patients receiving antibiotic treatment. In actual clinical practice, due to empirical diagnosis and misdiagnosis, the proportion of KD patients receiving antibiotic treatment is relatively high. According to relevant reports, more than half of the patients (54.3%) received antibiotic treatment before receiving KD standard treatment (25). The benefits of receiving antibiotic treatment for KD patients are still unclear. The unnecessary use of antibiotics has an impact on children's physical status and indicators, and increases the risk of side effects (26).

The patients enrolled in this study received antibiotics, mainly penicillin sodium (50IU), azithromycin dry suspension (0.1 g), and amoxicillin and clavulanate potassium tablets (0.23 g), and subsequently received IVIVG (intravenous immunogloblin, 2.5 g). According to some recommendations, the dose of IVIVG was suitbale (27). This study compared the differences in risk factors associated with the usage of antibiotics. Immature granular cell percentage and total protein were independent risk factors for KD with CAL among those who used no antibiotics, which exhibited a different pattern compared with the data setignored antibiotic use (all cases: immature granular cell percentage, total protein, and chlorine).

Total protein changes are related to the intensity of inflammation. The greater the intensity of inflammation, the longer the duration of fever, which is associated with the unresponsiveness of IVIG treatment and, therefore, an increased incidence of CAL (28). Suzuki et al. found that water retention in the acute phase of Kawasaki disease may be a risk factor for CAL (29), and that water retention causes changes in the patient's ion concentration. The results of their analyses were consistent with our findings that total protein and chlorine were independent risk factors for KD with CAL.

These analysis results of independent risk factors, on the one hand, clarified new independent risk predictors for KD with CAL, and on the other hand, the differences in factors also suggest that the consideration of antibiotic use may affect the final clarity of independent risk factors.

When comparing KD patients with or without antibiotics usage, albumin and globulin have significant differences in all cases; Total protein, albumin and globulin have significant differences in cases without CAL, indicating antibiotics can affect a patient's clinical indicators. In addition, albumin and globulin had been reported to predict KD with CAL (1, 4). This result further suggests that the use of antibiotics in the early stage of KD may affect the prediction and diagnosis, which may potentially increase the risk of CAL in KD, and it is important to consider antibiotics when exploring independent risk factors.

This study does have several limitations, including a small number of KD patients with a limited case number of antibiotics usage in KD patients, and it was a retrospective study, increasing the possibility of bias in interpreting the results. There is no Z score available now for children in China. The echocardiogram diagnosis of CALs was made by measuring the age-specific internal lumen diameter.

## Conclusion

This study has clarified that the independent risk factors for predicting CAL formation in KD patients without antibiotic usage include dimmature granular cell percentage and total protein. The most obvious finding to emerge from this study is that the use of antibiotics affected physiological indicators and the prediction and diagnosis of KD patients with CAL. However, this research has thrown up many questions in need of further investigation.

## Data Availability Statement

The original contributions presented in the study are included in the article/supplementary material, further inquiries can be directed to the corresponding author/s.

## Ethics Statement

The studies involving human participants were reviewed and approved by the Institutional Review Board of Baoan Women's and Children's Hospital, Shenzhen, China, with IRB No. LLSCHY2021-1-07. Written informed consent from the participants' legal guardian/next of kin was not required to participate in this study in accordance with the national legislation and the institutional requirements.

## Author Contributions

GZ and WZ conceptualized and designed the study. SL conceptualized the analysis for this article. WZ and SL drafted the manuscript and revised each version of the manuscript. HK conceptualized the analyses for this article. TZ and WZ supervised the data analyses and reviewed the manuscript. TZ contributed to the interpretation of the study findings. All authors participated in team discussions of data analysis and approved the final manuscript.

## Funding

This study was supported by the Baoan District Medical and Health Basic Research Project (2021JD054).

## Conflict of Interest

The authors declare that the research was conducted in the absence of any commercial or financial relationships that could be construed as a potential conflict of interest.

## Publisher's Note

All claims expressed in this article are solely those of the authors and do not necessarily represent those of their affiliated organizations, or those of the publisher, the editors and the reviewers. Any product that may be evaluated in this article, or claim that may be made by its manufacturer, is not guaranteed or endorsed by the publisher.
